# The Relationship between Social Anhedonia and Perceived Pleasure from Food—An Exploratory Investigation on a Consumer Segment with Depression and Anxiety

**DOI:** 10.3390/foods11223659

**Published:** 2022-11-16

**Authors:** Nikoline Bach Hyldelund, Derek Victor Byrne, Raymond C. K. Chan, Barbara Vad Andersen

**Affiliations:** 1 Food Quality Perception and Society Team, iSense Lab, Department of Food Science, Faculty of Technical Sciences, Aarhus University, 8000 Aarhus, Denmark; 2 Sino-Danish College (SDC), University of Chinese Academy of Sciences, Beijing 101408, China; 3 Neuropsychology and Applied Cognitive Neuroscience Laboratory, CAS Key Laboratory of Mental Health, Institute of Psychology, Chinese Academy of Sciences, Beijing 100101, China; 4 Department of Psychology, University of Chinese Academy of Sciences, Beijing 100101, China

**Keywords:** anhedonia, eating behavior, depression, anxiety, food pleasure, food reward

## Abstract

Anhedonia, the diminished ability to experience pleasure, is a key symptom of a range of mental and neurobiological disorders and is associated with altered eating behavior. This research study investigated the concept of anhedonia in relation to mental disorders and the perception of pleasure from food to better understand the link between anhedonia and eating behavior. A consumer survey (*n* = 1051), including the Food Pleasure Scale, the Chapman Revised Social Anhedonia Scale, the Patient Health Questionnaire, and the Generalized Anxiety Disorder scale, was conducted to explore the perception of pleasure from food among people with anhedonic traits. Comparative analyses were performed between people with symptoms of depression and/or anxiety and people with no symptoms of these conditions. A segmentation analysis was furthermore performed based on three levels of anhedonia: Low, Intermediate and High anhedonia. Thus, insights into how food choice and eating habits may be affected by different levels of anhedonia are provided for the first time. Our findings showed that the ‘Low anhedonia’ segment found pleasure in all aspects of food pleasure, except for the aspect ‘eating alone’. ‘Eating alone’ was, however, appreciated by the ‘Intermediate anhedonia’ and ‘High anhedonia’ segments. Both the ‘Intermediate anhedonia’ and ‘High anhedonia’ segments proved that their perceptions of food pleasure in general were affected by anhedonia, wherein the more complex aspects in particular, such as ‘product information’ and ‘physical sensation’, proved to be unrelated to food pleasure. For the ‘High anhedonia’ segment, the sensory modalities of food were also negatively associated with food pleasure, indicating that at this level of anhedonia the food itself is causing aversive sensations and expectations. Thus, valuable insights into the food pleasure profiles of people with different levels of anhedonia have been found for future research in the fields of mental illness, (food) anhedonia, and consumer behaviors.

## 1. Introduction

Imagine eating a beautifully constructed meal, cooked to perfection from high-quality foods of your specific preferences. Imagine eating this meal in an atmospheric restaurant with the person dearest to you. Now, imagine not perceiving the slightest degree of pleasure from this meal. This scenario seems almost impossible in today’s modern hedonic way of life; however, this might be what people with anhedonic traits experience. Anhedonia is a key symptom of a vast array of mental disorders and neurobiological diseases, ranging from mild anxiety to severe depression, schizophrenia, eating disorders, and Parkinson’s disease [1]. With the growing global prevalence of mental disorders, as well as obesity as a comorbidity, the further understanding of the symptoms of these health issues seems to be of paramount importance. In this research article, the concept of anhedonia in relation to mental disorders and eating behavior is investigated, so as to better understand the consequences of living with these disorders in terms of the perception of pleasure from food and meals. Accordingly, we shed light on how anhedonia can affect the perceived wellbeing from food and meals and, ultimately, individual eating behavior and health too.

### 1.1. ‘Anhedonia’ and Its Counterpart: ‘Reward’

The term anhedonia was first coined by the French psychologist Ribot in 1896 [2] as the ‘inability to experience pleasure’. Since then, numerous attempts have been made to reconceptualize anhedonia and to find ways to measure the concept according to both scientific disciplines and perspectives [3,4,5]. In particular, efforts have been made to further understand anhedonia and its counterpart, reward, by the subcomponents of ‘wanting’, ‘liking’, and ‘learning’ [6,7,8]. Hereby, reward and anhedonia are to be understood as constituting a cyclical process consisting of both an appetitive, consummatory, and satiated phase, each related to the aforementioned subcomponents [8]. In addition, each of these three subcomponents are also divided into a conscious and unconscious version. Thus ‘wanting’ is to be understood as the motivational processes of both incentive salience as well as conscious desires for an incentive or goal [3,9]. ‘Liking’ is the hedonic impact of a stimuli, which can both be an unconscious liking reaction and a conscious experience of pleasure [3,9]. Finally, the process of ‘learning’ can both be understood as associative conditioning and explicit predictions [3,9]. From this perspective, anhedonia may be a product of a deficiency in the processing of any of these subcomponents [4,8], just as any of these processes may also contribute to an overall sense of pleasure in the more positive outlook of reward.

Another conceptual view of anhedonia, which has proven popular in recent years, is that of the ‘Affective circumplex’ or ‘Positive affectivity’ [10,11]. ‘Positive affectivity’ stems from a more general theory of affective psychology, where all emotional experiences are organized within an affective space of two or more dimensions, for instance, positive or negative affect, or valence and arousal [10,11]. The dimensions by which all emotional experiences are differentiated by are not opposites but merely uncorrelated and individual spaces. In terms of understanding anhedonia within this conceptual framework, the notions of ‘positive affect’ and ‘negative affect’ become of great importance [10,12]. ‘Positive affect’ represents the extent to which a person is motivated for life’s experiences, whereas ‘negative affect’ represents the degree to which a person feels upset or unpleasantly aroused [10,11]. Thus, the feelings of ‘positive affect’, such as enthusiasm, energy, and motivation, are very much those that drive a person towards a reward. With this in mind, anhedonia may then be defined as involving low ‘positive affect’ rather than high ‘negative affect’ [10].

### 1.2. Anhedonia and the Link to Mental Disorders

Anhedonia features as one of the key symptoms within the definition of depression, other mood disorders, and schizophrenia [1,3,10]. Thus, anhedonia is also represented in the official diagnostic tools for Major Depressive Disorder (MDD), such as the Major Depressive Inventory [13] and the Diagnostic and Statistical Manual of Mental Disorders, Fifth Edition (DSM-5) [14]. The links between anhedonia and these disorders have been confirmed both by self-report measures, such as the Snaith–Hamilton Pleasure Scale [15] and the Chapman Physical and Social Anhedonia Scales [16], and by behavioral assessments in human studies with MDD and schizophrenia patients, as well as by neuroscientific experimental animal studies [1,3,17]. Anxiety disorders are likewise a group of mental disorders that have been associated with anhedonic traits, although not with the same level of severity as in MDD. Anxiety is commonly regarded as a predecessor of MDD, and it has been suggested that the mediating link between the two conditions is anhedonia [18,19]. When suffering from anxiety, experiences of positive reward may purposely be avoided, thus possibly also resulting in depressive symptoms over time [18]. Besides the well-documented link to depression, schizophrenia, and other mood disorders, anhedonia is also common among patients with Alzheimer’s disease, Parkinson’s disease, eating disorders, and substance use disorder [1].

### 1.3. Anhedonia and the Link to Eating Behaviour

For most people, eating is highly related to a sense of satisfaction and pleasure, and as such may be pivotal in maintaining wellbeing in a busy daily life. Nonetheless, a varied range of health conditions have been shown to affect appetite in a way that is not related to hunger or satiety sensations but the more affective side of eating. Eating disorders constitute an obvious example of such a condition. Eating disorders have consistently been related to both general and social anhedonia; however, depending on the nature of the eating disorder, anhedonic traits and reward sensitivity will be expressed in different eating patterns [20,21]. For instance, it has been found that reduced sensitivity to food pleasure will facilitate food avoidance in people with anorexia nervosa, whereas heightened sensitivity will lead to the overconsumption of highly palatable foods in people with bulimia nervosa or binge-eating disorder [20,21,22]. Both a reduced and a heightened sensitivity are considered an imbalance in reward perception that may lead to anhedonic traits, which is why binge-eating disorder has gained increasing attention in recent years, as anhedonia is believed to be an antecedent of this specific condition [22]. Thus, it has been suggested that people with binge-eating disorder, similar to people suffering from drug abuse, experience abnormally high levels of ‘wanting’ when encountering food, even in instances where the food is not particularly liked by the person [21]. As anhedonia is thought to arise from dysfunctions in the reward system, wherein the anticipation of a reward, the consumption of rewards, and the learning processes of reward are especially affected, it is possible that people with anhedonia have an increased drive to pursue comfort and pleasure from primary reinforcers, such as food, so as to compensate for these reward deficits [5,7,22]. Thus, affective states may temporarily be improved; however, the risk of developing binge-eating disorder and gaining weight also increases [20,22].

On a similar—although less severe—note, comfort eating or stress-induced eating can also be regarded as eating patterns that to some extent reflect anhedonic traits. The mechanism here very much resembles that of binge-eating disorder, as it is believed that highly palatable food is pursued and used as a means of comfort by people who feel stressed, regardless of whether the food elicits hedonic value during consumption [23,24,25,26]. A recent study found that people suffering from chronic stress indeed show signs of anhedonia and report changes in appetite, the perception of food pleasure, and eating behavior [27]. In addition, it was discovered that even short bouts of acute psychosocial stress have the potential to alter individuals’ food preferences by the specific reward mechanism of implicit wanting, leading to the choice of highly palatable foods [28]. Thus, it can be argued that people suffering from stress, even in short temporary events, likewise experience symptoms of anhedonia [27,28].

Finally, a comprehensive amount of research has investigated the relationship between anhedonia, weight gain, and obesity, as both anhedonia and obesity are tightly linked to MDD [19,22,26,29,30]. Mansur et al. (2019) concluded from a randomized, controlled study that overweight/obesity significantly moderated the association between MDD and the willingness to make an effort for a reward, specifically as a consequence of an increased anticipatory reward response [30]. Likewise, Ibrahim et al. (2016) found that higher anhedonic scores predicted long-term weight gain [29]. Thusly, a dysfunctional reward system may lead to metabolic disturbances too. However, how these ‘disturbances’ are experienced and come to be expressed by the affected person in relation to food and eating is yet to be investigated. To broaden our understanding of how anhedonia affects one’s perception of their quality of life, factors that are known influence the wellbeing of everyday life need attention. For many people, food is such a factor, as we eat multiple times a day, and often not for solely physiological reasons. Thus, further investigations into the link between anhedonic traits, mental health, and the pleasure perceived from food is pivotal in terms of both understanding what anhedonic people experience in relation to food and eating as well as to be able to help and alleviate this condition in the future. With this research paper, we wish to give an account of which drivers people with anhedonic traits experience as important when perceiving pleasure from food and meals. Thus, insights into how food choice and eating habits may be affected by different levels of anhedonia are provided for the first time.

### 1.4. Aims

The purpose of this article is to give an explorative account of how anhedonic traits, as exemplified by people with symptoms of major depressive disorder and/or general anxiety disorder, and the perception of pleasure from food may be related and influence each other. More specifically, the objectives of the current study were as follows:

- To explore whether people with symptoms of depression and anxiety as an example of a consumer group with anhedonic traits perceive pleasure from food differently than people without these symptoms;
- To explore whether segments defined by different degrees of social anhedonia likewise have different food pleasure profiles.

It was hypothesized that people with anhedonic traits would show a narrower range of aspects from which they perceive pleasure from food than people not displaying anhedonic traits. Likewise, it was assumed that the anhedonic group would generally assign lower importance to food pleasure when compared to a non-anhedonic group. In addition, it was hypothesized that different levels of anhedonia would manifest in different food pleasure profiles, i.e., with differences in the elements mainly contributing to pleasure from food and eating experiences, as anhedonia has proven to influence people in different ways, leading to different behavioral patterns [20,22,27,30].

## 2. Materials and Methods

To fulfill the aims and specific objectives of the study, a consumer survey was conducted among different consumer groups with presumed anhedonic traits, namely, people suffering from depression and anxiety. Well-known and validated scales were utilized to assess mental health states as well as social anhedonia levels. Thus, the nine-item Patient Health Questionnaire (PHQ-9) was used to assess symptoms of depression [31], the seven-item Generalized Anxiety Disorder (GAD-7) to assess symptoms of anxiety [32], and the Chapman Revised Social Anhedonia Scale (RSAS) to evaluate levels of social anhedonia [16]. These results were then combined with results of the Food Pleasure Scale, a newly developed research tool designed specifically for assessing drivers of pleasure derived from food and meals on an individual level [33]. Thus, both the first and second objectives of the study were met.

### 2.1. Data Collection

The consumer survey was administered in an online format to be completed in an at-home setting and was completed by a Chinese sample. To set up and distribute the questionnaire, the www.wjx.cn (accessed on 2 November 2021) online questionnaire platform [34] was used. The questionnaire itself was designed based on the items included in the original conceptual framework for the Food Pleasure Scale [33]. Figure S1 shows the 21 items included in this research. The FPS consists of two sections: first, the respondents were asked whether they generally experienced pleasure from each of the 21 items of the FPS when eating food. Next, they were asked to rate each item in terms of how important that item was to their sense of pleasure when eating food. They rated each item on a 5-point scale, where ‘1’ = ‘Not at all important’ and ‘5’ = ‘Extremely important’. A previously back-translated Chinese version of the FPS was utilized to ensure linguistic consistency and comprehension of the scale [35]. In addition, the questionnaire included socio-demographic questions, namely, the PHQ-9 [31], the GAD-7 [32], and the RSAS [16], and they were presented to the respondents in said order. Furthermore, the items within each scale were presented in a fixed order.

To evaluate current symptoms of major depressive disorder in the respondents, the PHQ-9 questionnaire was utilized. The PHQ-9 questionnaire comprises nine different diagnostic symptom criteria by which major depressive disorder is diagnosed, which have proven to be able to detect depressive disorder as well as its severity [32,36,37]. Furthermore, the PHQ-9 has been translated into various languages, including Chinese, and has been validated for screening of depressive disorders in many different ethnicities [36,38,39]. The PHQ-9 consists of nine questions, each representing the nine criteria for diagnosis of Major Depressive Disorder. Respondents were asked to rate the frequency of the depressive symptoms they have experienced in the 2 weeks prior to answering the questionnaire. This was performed using a 4-point nominal scale, where ‘0’ = ‘Not at all’, ‘1’ = ‘Several days’, ‘2’ = ‘More than half of the days’, and ‘3’ = ‘Nearly every day’. Ratings were then summed to yield a final score ranging from 0–27, with specific cut-off values determined as 0–4: No depression, 5–10: Mild depression, 11–15: Moderate depression, 16–20: Moderately severe depression, and 21–27: Severe depression [36,40]. Respondents with scores ranging from 11–27 were considered as suffering from depression in this study.

In addition to the PHQ-9, the GAD-7 questionnaire was utilized to evaluate symptoms of yet another mood disorder associated with anhedonia, namely, general anxiety disorder. The GAD-7 measures generalized anxiety disorder by seven different items, which altogether reflect the official symptom criteria for generalized anxiety disorder [32,41,42]. Respondents were asked to rate how often, during the last 2 weeks, they were bothered by each symptom. The response options were ‘0’ = ‘not at all’, ‘1’ = ‘several days’, ‘2’ = ‘more than half the days’, and ‘3’ = ‘nearly every day’. Like the PHQ-9, an individual total score was summed (range 0–21) and specific cut-off values were determined as 0–4: Minimal anxiety, 5–9: Mild anxiety, 10–14: Moderate anxiety, and 15–21: Severe anxiety [32,42,43]. Only respondents with a GAD-7 score above 10 were considered to be suffering from anxiety in this study.

The RSAS measures insufficiencies in the ability to experience pleasure from non-physical stimuli such as other people, talking, and exchanging expressions of feelings [16,44,45]. In this study, the RSAS was used as the basis for a segmentation analysis including a profiling of each segment, thus providing insights into how different levels of social anhedonia are reflected in relation to perception of food pleasure. The RSAS consists of 40 true–false questions addressing antisociality and indifference to others, assessed by measuring interpersonal pleasure. The scale has been proven to predict future development of schizophrenia-spectrum disorders [16,44]. A high total RSAS score indicates a lower capacity to feel pleasure and thereby greater level of anhedonia [16,44]. All participants were classified into three groups based on their scores on the Chinese version of the Chapman Revised Social Anhedonia Scale [16,44]. We used the means and standard deviations of the RSAS (Mean = 10.72; SD = 6.46) from our earlier cohort of 2167 Chinese college students as the reference range in this study [46,47]. According to the cut-off score criteria of the RSAS in previous studies [46,47], the individuals with an RSAS score higher than 1.5 SD above the mean score (cut off score ≥ 20) were classified as high social anhedonia group (*n* = 154), while participants with RSAS score lower than the mean score (cut off score ≤ 11) were classified as the low social anhedonia group (*n* = 537). Moreover, to maximize the use of the entire sample rather than only using the extreme groups’ perspective, we referred to previous research [48], categorizing the remaining participants lying in between the high and low levels of social anhedonia as intermediate level of social anhedonia group (*n* = 360). The internal reliability of the RSAS results of the sample was tested via Cronbach’s alpha scores and was found to be 0.67 for the entire sample.

Lastly, socio-demographic information was collected and consisted of the following variables: ‘gender’, ‘age’, ‘educational level’, ‘father’s age and educational level’, ‘mother’s age and educational level’, ‘income level of total family’, ‘personal history with mental illness’, and ‘family history with mental illnesses’.

### 2.2. Participants and Recruitment

In this study, respondents were mainly students at universities from a total of 23 different Chinese regions in mainland China. The respondents participated through an online advertisement, where they responded to a hyperlink leading to the questionnaire. A total of 1138 respondents completed the questionnaire. Only people above the age of 18 were included in the data analysis, which resulted in a final sample of 1051 respondents (676 males and 375 females) with a mean age of 21.90 ± 4.10 (18–50 years). An overview of the characteristics of the respondents can be seen in Table 1.

Participants were recruited through an open advertisement with an online link with a set of questionnaires during 15 September 2021 to 5 October 2021. All participants gave written consent online. This study was approved by the Ethics Committee of the Institute of Psychology, Chinese Academy of Sciences (Protocol number: H20041).

### 2.3. Data Analysis

Data analysis consisted of two parts so as to comply with the objectives of the study. First, each respondent was assigned to a group based on their health status as assessed by the PHQ-9 and GAD-7. The PHQ-9 was utilized to evaluate levels of major depressive disorder, whereas the GAD-7 was used to assess level of general anxiety in each respondent. All respondents who had a score ≥11 on the PHQ-9 and/or ≥10 on the GAD-7 were regarded as belonging to the ‘Depression/anxiety’ group (DEPANX), while all other respondents were regarded as healthy, and thus were assigned to the ‘non-DEPANX’ group. Thus, *n* = 349 (33%) belonged to the ‘DEPANX’ group, while *n* = 702 (67%) respondents were in the ‘non-DEPANX group. Descriptive analyses were conducted based on these two groups to investigate the differences between them. Mean (±SD) or median values (IQR) were calculated for each variable for each consumer group, and results were illustrated by either bar plots based on the mean values or by stacked bar plots showing the distributions of answers by the two groups. Normal distribution of the data was checked by the Shapiro–Wilk test, and subsequent statistical tests were chosen accordingly. Wilcoxon’s signed rank test was utilized to detect significant differences between the two groups regarding numerical vales, whereas Chi^2^ tests were used for the categorical variables. All data analyses were conducted in R Studio©, version 2022.02.3 + 492 (Boston, MA, USA) [49]. Statistical significance was set to α < 0.05 for all calculations.

#### Segmentation and Profiling of Consumer Segments According to Anhedonic Levels

The second step of the data analysis was performed to obtain more nuanced insights in terms of the relationship between specific anhedonic levels and perception of food pleasure. The respondents were thus re-assigned to three different groups based on the specified cut-off values of the RSAS. Thus, the ‘Low anhedonic’ group consisted of respondents with an RSAS score of 0–10 (*n* = 537, 51%), the ‘Intermediate anhedonic’ group of people with a score of 11–20, (*n* = 360, 34%), and the ‘High anhedonic’ group with a score of 21–40 (*n* = 154, 15%). Mean (±SD) or median values (IQR) were calculated for each variable for each consumer group, and selected results were illustrated by appropriate plots. Consequently, a segmentation analysis based on these three groups was performed, first by detecting differences between the groups by Chi^2^ tests for categorical variables or by Kruskal–Wallis tests for numerical values. For profiling of the consumer segments, multivariate analysis by binary logistic regression and odds ratios were calculated for all variables including lower and upper levels for 95% confidence intervals. Thus, segment membership was used to define categorical dependent variables, whereas the independent variables of the models included the sociodemographic and mental health variables, as well as the results of the Food Pleasure Scale. All calculations of odds ratios were adjusted for age and gender, as these two variables were believed to be possible confounders.

All data analyses were conducted in R Studio©, version 2022.02.3 + 492 (Boston, MA, USA) [49]. Statistical significance was set to α < 0.05 for all calculations. For 0.05 < α < 0.08, results were reported as ‘trending’ towards a significant difference [50,51].

## 3. Results

### 3.1. Description of Respondents According to Mental Health Status

**Figure 1:**
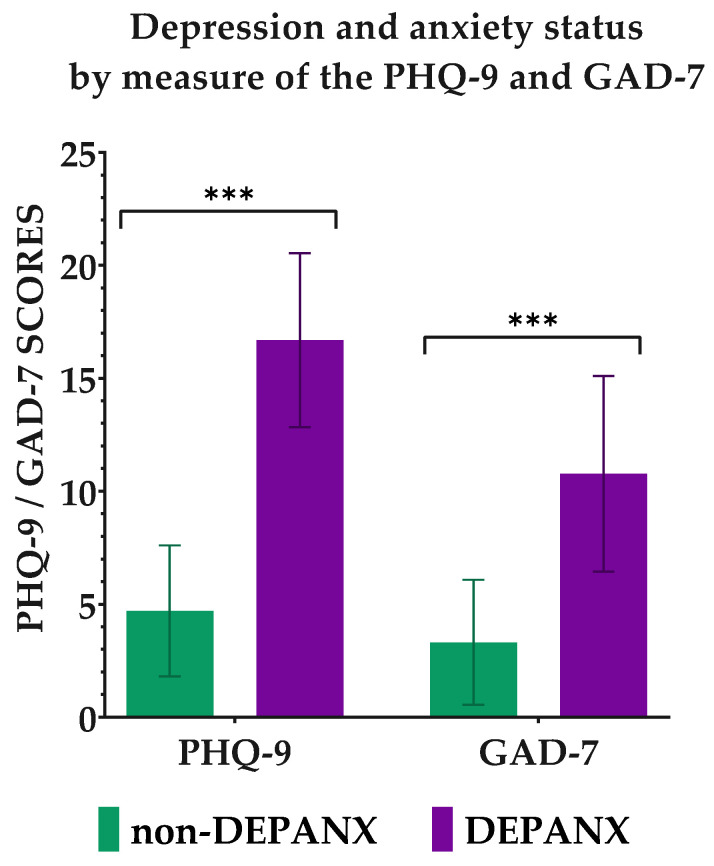
Depression and anxiety status by measure of the PHQ-9 and GAD-7. Results are shown for the two groups ‘non-DEPANX’ (*n* = 702) and ‘DEPANX’ (*n* = 349). Stars indicate level of significance of p-values. ***: *p* < 0.001.

The two groups (‘DEPANX’ and ‘non-DEPANX’) proved to be quite similar in terms of the socio-demographic variables. Only gender showed significant differences in distribution, as the ‘non-DEPANX’ group consisted of 68% males and 32% females, whereas the ‘DEPANX’ group was 57% male and 43% female. All other sociodemographic variables showed results similar to the whole group, as depicted in Table 1. In relation to mental health, naturally, the groups were diversified with respect to all variables. Thus, the ‘DEPANX’ group scored higher on both the PHQ-9 (*p* < 0.001), with a mean score of 16.68 ±3.85, and the GAD-7 (*p* < 0.001), with a mean score of 10.77 ± 4.33. A PHQ-9 score ≥ 11 is considered a sign of either moderate (11–15), moderately severe (16–20), or severe (21–27) depression, whereas a GAD-7 score ≥ 10 is considered as moderate (10–14) or severe (15–21) anxiety. In comparison, the ‘non-DEPANX’ group scored 4.70 ± 2.90 and 3.31 ± 2.77 on the PHQ-9 and GAD-7, respectively. When asked about a family history of mental illness diagnosis, both groups claimed to have no occurrences of mental illnesses in their families. However, the ‘DEPANX’ group had a slightly higher frequency of people who were unsure about this question (*p* = 0.035). Figure 1 illustrates the differences regarding mental health between the two groups.

### 3.2. Perception of Pleasure by Healthy vs. Depression/Anxiety Consumers

**Figure 2:**
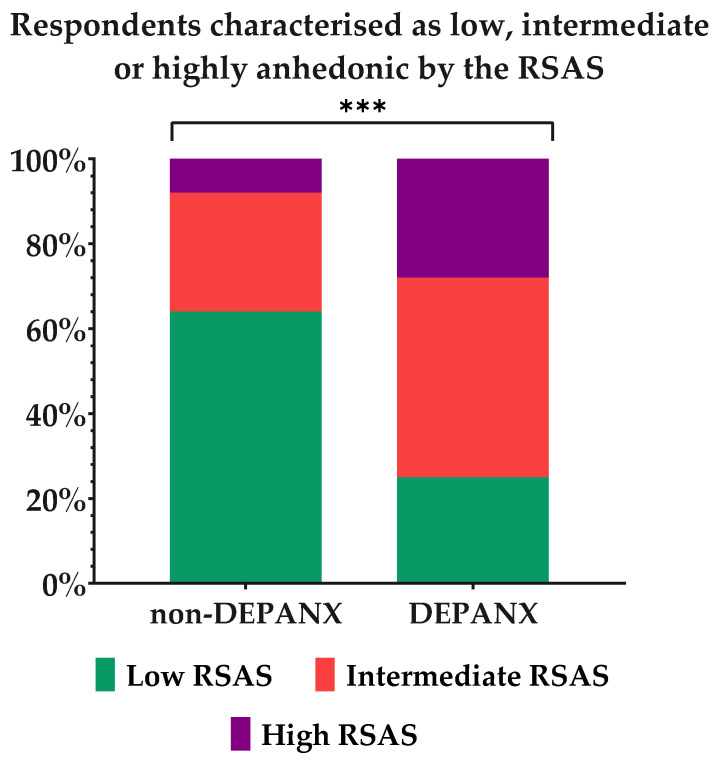
Social anhedonia levels in each group by measure of the Revised Social Anhedonia Scale. Results are shown for the two groups, ‘non-DEPANX’ (*n* = 702) and ‘DEPANX’ (*n* = 349). Asterisks indicate level of significance of *p*-values. ***: *p* < 0.001.

Social anhedonia was measured by the RSAS, where a score of 0–10 = Low social anhedonia, 11–20 = Mild social anhedonia, and 20–40 = High social anhedonia. Here, the two groups again proved to be different, as the ‘non-DEPANX’ group had a mean score of 10.28 ± 6.76, whereas the DEPANX group scored 17.01 ± 7.33 on average. Figure 2 shows the distribution within the two groups in terms of the level of social anhedonia. From this figure, there is a clear difference in the distribution, as the ‘non-DEPANX’ group has a larger group of people with low social anhedonia (*p* < 0.001), and, oppositely, the DEPANX group has relatively more with intermediate and high social anhedonia (*p* < 0.001 for both levels). These results thereby indicate that depression and anxiety may well be correlated with traits of social anhedonia.

**Figure 3:**
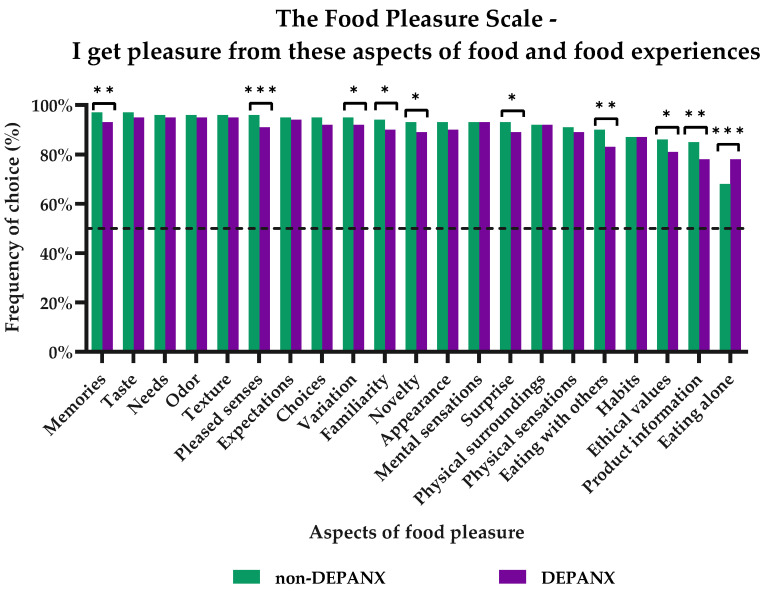
Subjective perception of pleasure from food and food experiences by the 21 different pleasure aspects of the Food Pleasure Scale. Results are shown for the two groups: ‘non-DEPANX’ (*n* = 702) and ‘DEPANX’ (*n* = 349). Stars indicate level of significance of *p*-values. *: *p* < 0.05, **: *p* < 0.01, ***: *p* < 0.001. Dotted line marks 50% frequency.

The participants were all asked to choose the aspects of food and meals from which they receive pleasure among the twenty-one different aspects of the Food Pleasure Scale. Figure 3 provides an overview of the distribution of frequencies of choice in both groups. More than 60% of both groups claimed to receive pleasure from all aspects of the FPS. However, variations in frequency could be detected, both in terms of the most chosen aspects for the whole sample as well as for the two groups. In general, the sensory aspects of Taste, Odor, Texture, and Pleased senses were among the most-chosen aspects providing pleasure with food-related experiences. The aspects of fulfilling one’s needs and expectations as well as memories around food were likewise found among the topmost chosen aspects. However, differences between the two groups with respect to the frequency of choice could be seen for the aspects of pleased senses and memories, with the ‘DEPANX’ group choosing both aspects less frequently than the ‘non-DEPANX’ group. Among both groups, the smallest number of people experienced pleasure from the aspects of eating alone, having product information, fulfilling ethical values, maintaining food-related habits, and eating with others. Interestingly, significant differences were found for both eating with others and eating alone, wherein eating with others was chosen to provide pleasure more often in the ‘non-DEPANX’ group, while eating alone was chosen to provide pleasure to more respondents in the DEPANX group. There were likewise differences regarding the frequency of choice for product information and ethical values, with both chosen less frequently by the DEPANX group. Lastly, pleasure from the aspects of variation, familiarity, novelty, and surprise were all chosen less frequently by the DEPANX group (*p*-values ranging from 0.019–0.035), thus indicating that fewer people in this group receive pleasure from these aspects.

Together, these results indicate that people with depression and/or anxiety have different hedonic profiles compared to people without depression and/or anxiety, both on a more general level as well as in relation to specific food pleasure aspects. A closer examination of how the different levels of anhedonia are reflected in the perception of food pleasure will now be pursued to ensure a deeper understanding of what drives pleasure from food.

### 3.3. Three Levels of Social (An)Hedonia

By dividing the sample into three groups based on their individual RSAS scores, a segmentation analysis incorporating the profiling of each group by multivariate regression and calculations of odds ratios was possible. Thus, the groups consisted of the ‘Low anhedonia’ group (*n* = 537, 51%), with an RSAS score ranging between 0–10; the ‘Intermediate anhedonia’ group (*n* = 360, 34%), with an RSAS score of 11–20; and the ‘High anhedonia’ group (*n* = 154, 15%), with an RSAS score of 20–40. Table 2 shows the characteristics of each segment. In general, no differences were detected between the three segments regarding socio-economic variables. There were differences in terms of the family history of mental illnesses (*p* = 0.023), with increasing frequencies of being unsure on this matter for the ‘Intermediate’ and ‘High’ anhedonia groups. Likewise, the Chi^2^-tests showed differences between the groups in terms of the number of people who fell within the boundary of having signs of depression determined by the PHQ-9 (*p* < 0.001) and anxiety by the GAD-7 (*p* < 0.001), again with larger frequencies for the ‘Intermediate’ and ‘High’ anhedonia groups. Finally, the results of the RSAS also proved to be significantly different (*p* < 0.001), with mean values of 6.48 ± 3.03, 15.82 ± 2.74, and 25.83 ± 4.41 for the ‘Low’, ‘Intermediate’, and ‘High’ anhedonia groups, respectively.

**Figure 4:**
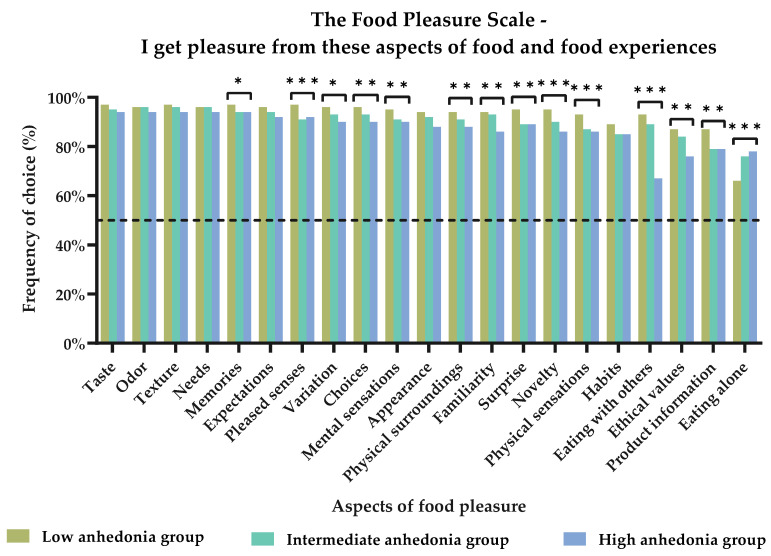
Subjective perception of pleasure from food and food experiences by the 21 different pleasure aspects of the Food Pleasure Scale. Results are shown for the three anhedonia groups: ‘Low anhedonia’ (*n* = 537), ‘Intermediate anhedonia’ (*n* = 360), and ‘High anhedonia’ (*n* = 154). Stars indicate level of significance of *p*-values. *: *p* <0.05, **: *p* < 0.01, and ***: *p* < 0.001. Dotted line marks 50% frequency.

In addition, the three segments proved to have different profiles in relation to the perception of food pleasure as measured by the FPS. Thus, the order of frequencies of the 21 FPS aspects from which the three segments receive pleasure differed in many instances. Figure 4 illustrates the aspects chosen most in the three segments, as well as where significant differences could be detected. In general, all aspects were chosen by at least 65% of the participants in each segment, with ‘Taste’, ‘Odor’, ‘Texture’, ‘Needs’, and ‘Memories’ as the top five most-chosen aspects. For the ‘Low anhedonia’ segment, all aspects were chosen by at least 87%, meaning that the majority perceived pleasure from all aspects of food pleasure. However, one exception was the aspect of ‘Eating alone’, which was chosen to provide pleasure to 66% of members in this group. In the ‘Intermediate anhedonia’ group, almost all aspects were chosen by at least 79% of the group; however, the aspect of ‘Eating alone’ was chosen by only 76% of the group. Finally, in the ‘High anhedonia’ group, all aspects were chosen by at least 76% of the respondents; however, ‘Eating with others’ was chosen only to provide pleasure to 67%.

In the following paragraphs, a more detailed characterization of each segment will be given based on the significant results of the multivariate regression models. For a complete overview of these results, see Table S1.

#### 3.3.1. The ‘Low Anhedonia’ Group

The ‘Low anhedonia’ group was the largest of the three segments with 51% of the respondents. This group had a slightly increased likelihood of having parents with more extensive education (Father’s education, OR: 1.05 and *p* = 0.017; Mother’s education, OR: 1.03 and *p* = 0.035), as well as a decreased likelihood of 69% (OR: 0.31 and *p* = 0.010) of belonging to the lowest income group. The group also had decreased likelihoods of suffering from depression and/or anxiety (PHQ-9—OR: 0.81 and *p* < 0.001; GAD-7—OR: 0.82 and *p* < 0.001).

**Figure 5:**
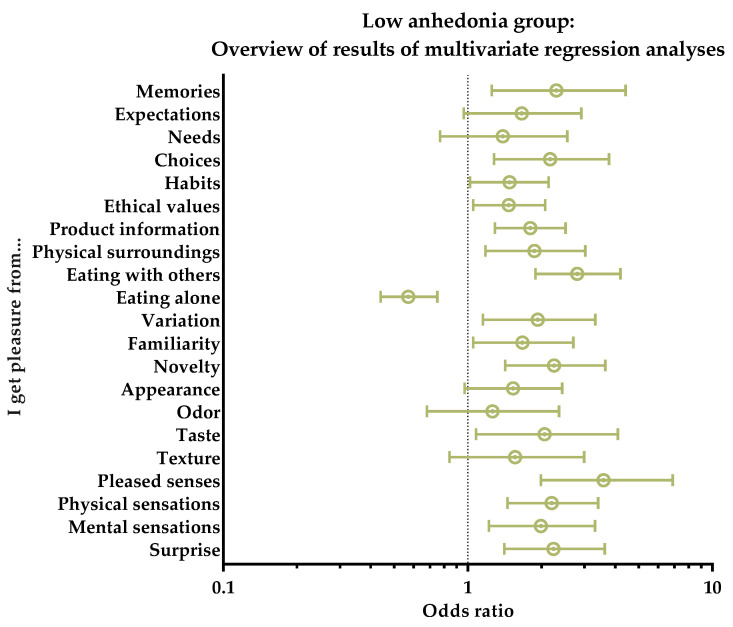
Low anhedonia group. Overview of results of multivariate regression analyses for the subjective perceptions of the 21 different pleasure aspects of the Food Pleasure Scale. Results are shown as odds ratios, including interquartile ranges. An odds ratio > 1 indicates an increasedlikelihood of choice, whereas odds ratio < 1 indicates a decreased likelihood of choice.

When asked about which of the 21 different aspects of the FPS the group received pleasure from, the group consistently had increased likelihoods of receiving pleasure from almost all aspects of the scale. Only the aspect of ‘Eating alone’ showed a decreased likelihood of 43% (*p* < 0.001). All other aspects had odds ratios ranging from 1.26–3.59, wherein ‘Pleased senses’ had the highest odds ratio (OR: 3.59, *p* < 0.001), followed by ‘Eating with others’ (OR: 2.80, *p* < 0.001), ‘Memories’ (OR: 2.30, *p* = 0.009), ‘Novelty’ (OR: 2.25, *p* < 0.001), and ‘Surprise’ (OR: 2.24, *p* < 0.001), to name a couple. The aspects of ‘Needs’, ‘Odor’, and ‘Texture’ did not yield significant results; thus, these three aspects did not contribute to the profiling of this group. Figure 5 gives an overview of the results described above.

In terms of rating the importance of each aspect to their sense of pleasure from food, again, almost every aspect had an increased likelihood of being rated very important. This included the aspects of ‘Memories’ (OR: 1.49, *p* = 0.011), ‘Expectations’ (OR: 1.50, *p* = 0.009), ‘Needs’ (OR: 1.54, *p* = 0.007), ‘Choices’ (OR: 1.67, *p* < 0.001), ‘Habits’ (OR: 1.49, *p* = 0.006), ‘Product information’ (OR: 1.60, *p* = 0.001), ‘Physical surroundings’ (OR: 1.33, *p* = 0.049), ‘Eating with others’ (OR: 1.81, *p* < 0.001), ‘Variation’ (OR: 1.50, *p* = 0.014), ‘Familiarity’ (OR: 1.45, *p* = 0.014), ‘Novelty’ (OR: 1.58, *p* = 0.001), ‘Taste’ (OR: 1.58, *p* = 0.015), ‘Texture’ (OR: 1.67, *p* = 0.008), ‘Pleased senses’ (OR: 2.03, *p* < 0.001), and ‘Mental sensations’ (OR: 1.62, *p* = 0.004). On a similar note, many of the aspects had a decreased likelihood of being rated as ‘not very important’. This was true for the aspects of ‘Ethical values’ (OR: 0.68, *p* = 0.042), ‘Physical surroundings’ (OR: 0.51, *p* = 0.005), ‘Eating with others’ (OR: 0.51, *p* = 0.001), ‘Appearance’ (OR: 0.60, *p* = 0.020), ‘Odor’ (OR: 0.51, *p* = 0.020), ‘Texture’ (OR: 0.42, *p* = 0.015), ‘Pleased senses’ (OR: 0.49, *p* = 0.040), ‘Physical sensations’ (OR: 0.37, *p* < 0.001), ‘Mental sensations’ (OR: 0.57, *p* = 0.037), and ‘Surprise’ (OR: 0.35, *p* < 0.001). Finally, the aspect of ‘Eating alone’, was the only aspect to have a decreased likelihood of being rated ‘very important’ (OR: 0.76, *p* = 0.061); however, this should be regarded as a ‘trending’ result, as the *p*-value was above 0.05.

#### 3.3.2. The ‘Intermediate Anhedonia’ Group

The second largest group of the three, the ‘Intermediate anhedonia’ group, consisted of 34% of all the respondents. This group had an increased likelihood of belonging to the lowest income group (OR: 3.26, *p* = 0.004) as the only sociodemographic variable to characterize the respondents. In relation to mental health, the group showed a slightly increased likelihood of 9% of showing symptoms of depression by scoring high on the PHQ-9 scale (OR: 1.09, *p* < 0.001). Moreover, it also had a 9% increased likelihood of scoring high on the GAD-7 (OR: 1.09, *p* < 0.001), thus also showing symptoms of anxiety.

**Figure 6:**
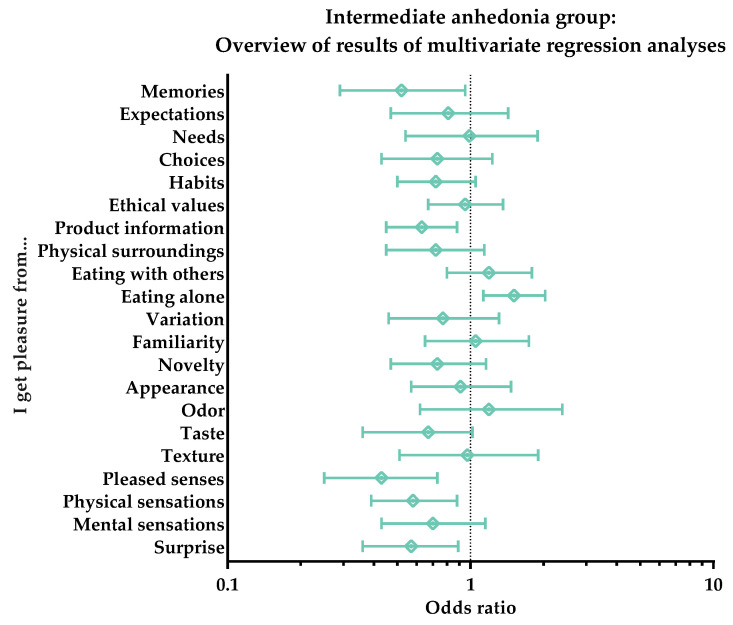
Intermediate anhedonia group. Overview of results of multivariate regression analyses for the subjective perception of the 21 different pleasure aspects of the Food Pleasure Scale. Results are shown as odds ratios, including interquartile ranges. An odds ratio > 1 indicates an increased likelihood of choice, whereas odds ratio < 1 indicates a decreased likelihood of choice.

The food pleasure profile of the ‘Intermediate anhedonia’ group showed significant results with respect to only a few of the aspects, and all of these had decreased likelihoods of bringing the respondents in the group pleasure. A low OR value was found for the aspects of ‘Product information’ (OR: 0.63, *p* = 0.007), ‘Physical sensations’ (OR: 0.58, *p* = 0.009), ‘Surprise’ (OR: 0.57, *p* = 0.014), ‘Memories’ (OR: 0.52, *p* = 0.031), and ‘Pleased senses’ (OR: 0.43, *p* = 0.002). Oppositely, ‘Eating alone’ was the only aspect with an increased likelihood of bringing this group pleasure, as the odds ratio for this aspect was 1.51 (*p* = 0.005). Figure 6 shows an overview of these results.

The odds ratios regarding the rating of the importance of each aspect yielded similar results. Again, only a few of the aspects showed significant results, and again all of these pointed towards decreased likelihoods of being important in terms of perceived pleasure. The following aspects all had decreased likelihoods of being rated ‘very important’ for the perception of pleasure in the ‘Intermediate anhedonia’ group: ‘Familiarity’ (OR: 0.74, *p* = 0.047), ‘Expectations’ (OR: 0.72, *p* = 0.042), ‘Habits’ (OR: 0.69, *p* = 0.015), ‘Eating with others’ (OR: 0.68, *p* = 0.012), ‘Product information’ (OR: 0.67, *p* = 0.009), ‘Taste’ (OR: 0.67, *p* = 0.037), ‘Memories’ (OR: 0.65, *p* = 0.007), ‘Mental sensations’ (OR: 0.65, *p* = 0.013), ‘Choices’ (OR: 0.64, *p* = 0.005), ‘Texture’ (OR: 0.51, *p* < 0.001), and ‘Pleased senses’ (OR: 0,48, *p* < 0.001). In addition, similar tendencies could be detected for a rating of ‘very important’ of the aspects of ‘Ethical values’ (OR: 0.76, *p* = 0.075), ‘Novelty’ (OR: 0.76, *p* = 0.071), ‘Physical sensations’ (OR: 0.76, *p* = 0.079), and ‘Variation’ (OR: 0.74, *p* = 0.070). In the same way, the aspect of ‘Surprise’ had an increased likelihood of being rated ‘not very important’ (OR: 1.63, *p* = 0.033) by the group.

#### 3.3.3. The ‘High Anhedonia’ Group

The smallest segment, the ‘High anhedonia’ group, made up 15% of the sample. This group had a tendency towards a higher likelihood of being female (OR: 1.38, *p* = 0.068). Similar to the ‘Intermediate anhedonia’ group, this group also had increased likelihoods of showing symptoms of depression and anxiety by scoring high on the PHQ-9 (OR: 1.19, *p* < 0.001) and GAD-7 (OR: 1.18, *p* < 0.001), respectively. In addition, there was a tendency of increasing uncertainty regarding a family history of mental illnesses in this group (OR: 2.18, *p* = 0.055).

**Figure 7:**
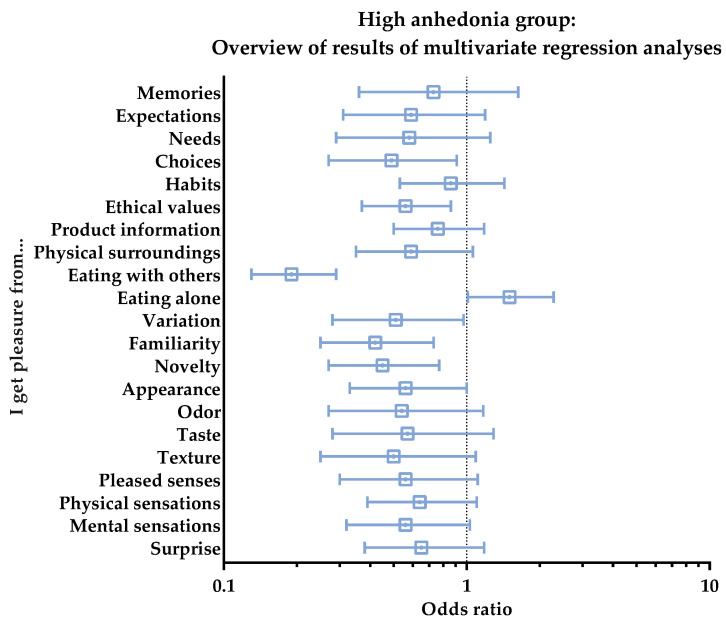
High anhedonia group. Overview of results of multivariate regression analyses for the subjective perception of the 21 different pleasure aspects of the Food Pleasure Scale. Results are shown as odds ratios, including interquartile ranges. An odds ratio > 1 indicates an increased likelihood of choice, whereas odds ratio < 1 indicates a decreased likelihood of choice.

The results of the multivariate regression analysis with respect to the individual aspects of the Food Pleasure Scale showed that only seven different aspects characterized this group. These aspects all had decreased likelihoods of giving pleasure to the group, and they were the aspects of ‘Eating with others’ (OR: 0.19, *p* < 0.001), ‘Familiarity’ (OR: 0.42, *p* = 0.001), ‘Novelty’ (OR: 0.45, *p* = 0.003), ‘Choices’ (OR: 0.49, *p* = 0.018), ‘Variation (OR: 0.51, *p* = 0.030), ‘Ethical values’ (OR: 0.56, *p* = 0.007), and ‘Appearance’ (OR: 0.56, *p* = 0.040). Furthermore, similar decreasing tendencies were detected for ‘Texture’ (OR: 0.50, *p* = 0.063), ‘Mental sensations’ (OR: 0.56, *p* = 0.051), ‘Pleased senses’ (OR: 0.56, *p* = 0.077), and ‘Physical surroundings’ (OR: 0.59, *p* = 0.067). Finally, the aspect of ‘Eating alone’ showed a tendency of a 50% increase in this group (OR: 1.50, *p* = 0.053). An overview of these results can be seen in Figure 7.

In terms of the importance ratings of each aspect, this segment was only positively characterized by one food pleasure aspect, ‘Eating alone’, which was the only aspect with an increased likelihood of being rated ‘very important’ at 77% (OR: 1.77, *p* = 0.010). All other aspects had either decreased odds ratios for being rated ‘very important’ or increased odds ratios for ‘not very important’. The aspects of ‘Texture’ (OR: 5.76, *p* < 0.001), ‘Pleased senses’ (OR: 3.30, *p* = 0.001), ‘Eating with others’ (OR: 2.84, *p* < 0.001), ‘Physical sensations’ (OR: 2.70, *p* < 0.001), ‘Surprise’ (OR: 2.31, *p* = 0.003), ‘Appearance’ (OR: 2.28, *p* = 0.003), ‘Choices’ (OR: 2.08, *p* = 0.010), ‘Memories’ (OR: 1.99, *p* = 0.037), and ‘Ethical values’ (OR: 1.76, *p* = 0.030) all had increased likelihoods of being rated ‘not very important’. Similar tendencies were detected for the aspects of ‘Variation’ (OR: 1.86, *p* = 0.056), ‘Expectations’ (OR. 1.84, *p* = 0.053), ‘Mental sensations (OR: 1.78, *p* = 0.068), and ‘Physical surroundings’ (OR: 1.69, *p* = 0.059). On a similar note, the following aspects had decreasing likelihoods of being rated ‘very important’: ‘Needs’ (OR: 0.62, *p* = 0.026), ‘Eating with others’ (OR: 0.57, *p* < 0.001), and ‘Novelty’ (OR: 0.66, *p* = 0.033). The aspect of ‘Physical surroundings’ (OR: 0.70, *p* = 0.078) likewise showed a tendency of a decreased likelihood of being rated ‘very important’ by this group.

## 4. Discussion

### 4.1. Differences in Food Pleasure Profiles: ‘DEPANX’ vs. ‘Non-DEPANX’

The results of the RSAS, as illustrated in Figure 2, showed that there were significantly larger proportions of respondents in the DEPANX group with either an intermediate or high anhedonic score than in the ‘non-DEPANX’ group. These results indicate that depression and anxiety are indeed correlated with social anhedonia, and thereby confirm the findings of prior studies [18,52]. Previous studies using the Chapman Social Anhedonia Scale have likewise also found positive correlations between high social anhedonia scores and major depressive disorder, schizophrenia spectrum disorders, general anxiety disorder, autism spectrum disorder, eating disorders, and type 2 diabetes [19,53,54,55,56]. Thus, these results inform us that social anhedonia can be seen as an influential factor in many health-related conditions, especially those related to mental health [53,54,57]. Likewise, the choice of the respondent sample has proven feasible in terms of exploring the relationship between anhedonic traits and the perception of food pleasure.

The results of the Food Pleasure Scale for both groups of respondents showed that differences could indeed be detected between the two groups (See Figure 3). When taking a closer look at the results regarding the frequency-of-choice ratings of each food pleasure aspect, it becomes evident that a higher proportion of the ‘non-DEPANX’ group in general receive pleasure from more aspects of the scale than in the DEPANX group. Thus, overall, a reduction in the variety of aspects was seen, as well as in the frequencies of choice for the ‘DEPANX’ group. Significant differences were also detected, with the ‘non-DEPANX’ group choosing the aspects of ‘Memories’, ‘Pleased senses’, ‘Variation’, ‘Familiarity’, ‘Novelty’, ‘Surprise’, ‘Eating with others’, ‘Ethical values’, and ‘Product information’ more frequently. These aspects of food and eating have all previously been characterized as ‘additional’ or ‘secondary’ aspects of a meal and not the food itself [27,35,58]. This indicates that people with mental health disorders focus on the more fundamental source of wellbeing and pleasure from food, that is, the intrinsic properties of the food, i.e., the sensory characteristics. Similar results were found in a previous study investigating the link between chronic stress and food pleasure [27]. One aspect proved to show the opposite picture, as the ‘DEPANX’ group chose ‘Eating alone’ significantly more than the ‘non-DEPANX’ group. Therefore, it seems that this specific aspect is where the two groups truly set each other apart. Belonging to the ‘DEPANX’ group implies scoring high on either the PHQ-9 or the GAD-7. As the link between depression, anxiety, and social anhedonia was cemented long ago [3,10,19], it is thus entirely reasonable that this is where the major difference is seen.

Although at first glance the differences detected between the two groups may seem most fascinating, the similarities between the two also reveal some interesting results. The sensory aspects of food and eating (‘Taste’, ‘Odor’, and ‘Texture’) were found among the topmost chosen aspects for both groups. Thus, these aspects all provided pleasure for both groups and thus attested to the fact that the intrinsic product characteristics of food may operate as the foundation for a pleasurable experience. These results mirror those found in previous studies by the current authors [27,28,35,58], as well as confirm findings in the literature in the field of food satisfaction and wellbeing, which have found a strong relation between the sensory properties of food and satisfaction and liking [59,60]. In addition, the aspects of fulfilling one’s ‘Needs’ and ‘Expectations’ and having ‘Choices’ were also among the most chosen for both groups. Another study on a Chinese sample also showed that these specific aspects were among the most important for the perception of food pleasure; however, the sensory aspects were also most important in this study [35]. Having one’s needs and expectations fulfilled from a meal may be an expression of two phenomena: for one, it could be related to the basic physiological needs of reducing hunger sensations as well as specific expectations towards the satisfactory level of the food [61,62]. However, it could also be related to the basic human needs of feeling safety and security, as described in Maslow’s theory of the ‘Hierarchy of Needs’ [63]. Nonetheless, these sensory-driven and cognition-driven aspects of food pleasure have proven to yield pleasure regardless of one’s mental health status.

### 4.2. The Effect of Different Degrees of Anhedonia on Food Pleasure Profiles

The segmentation analysis based on the three levels of social anhedonia yielded several results that were not entirely clear from the perspective of the division between the respondents with depression and/or anxiety and the respondents without symptoms of these conditions. Thus, performing the segmentation analysis provided more nuanced insights into how anhedonia is reflected in pleasure from food. The profile of the ‘Low anhedonia’ group depicted a segment that found pleasure not only in the more basic elements of food, such as the sensory aspects, but also from the more advanced aspects of food pleasure, such as novelty, surprises, and memories. Thus, this segment resembled in great detail the description of food pleasure profiles found in previous studies performed on ‘normal’ healthy adults [35,58]. This also reflects a group of people who have the surplus of mental resources required to pursue and expect pleasure from more complex aspects than just the sensory input. In addition, the aspect of ‘Eating alone’ is the only aspect of food pleasure that has a decreasing likelihood of contributing to pleasure in this segment. This underlines the fact that liking food and eating are considered social acts to many [59,64]. When focusing on the ‘Intermediate anhedonia’ group, differences in the effect of the anhedonia levels on food pleasure are evident. However, this group depicts a segment that has a somewhat ‘indistinct’ profile, with a clear indication that the secondary aspects of food pleasure—such as ‘product information’, ‘physical sensations’, ‘surprises’, ‘memories’, and ‘pleased senses’—are less likely to contribute to their sense of food pleasure. However, ‘eating alone’ was the only aspect that made a positive contribution to food pleasure in this group. Finally, the food pleasure profile of the ‘High anhedonia’ group demonstrated a complete reversal compared to the ‘Low anhedonia’ group. Like the ‘Intermediate’ group, ‘Eating alone’ is the only aspect that provided pleasure, and with an even higher likelihood, as an increase of 50% was seen here. This increase seems reasonable as the segments were defined by their social anhedonia level. Furthermore, almost all the other aspects had decreased likelihoods of contributing to food pleasure for this segment. This was especially true for the more complex aspects; thus, the food pleasure profile of the ‘High anhedonia’ group greatly resembles that of the ‘Intermediate anhedonia’ group, but it is amplified both in terms of the number of aspects affected and in relation to the degree of decrease in the likelihoods detected by the OR levels. Moreover, the basic drivers of pleasure, the food’s appearance and texture, were also negatively affected in this segment. This indicates that this level of anhedonia has reached a point where the food itself is starting to cause aversive sensations and expectations. This is interesting, as social anhedonia and eating disorders have previously been linked [20,22]; therefore, these results may, to some extent, confirm this link or at least point towards a clarification of how highly anhedonic people perceive pleasure from food and eating experiences.

An unexpected result of the segmentation analyses consisted of the differences detected regarding social-economic status (SES), as no differences could be detected between the three groups with respect to these variables in the initial Chi^2^ tests. The ‘Low anhedonia’ group had an increased likelihood of having parents with more extensive educations and a decreased likelihood of belonging to the low-income group, whereas the ‘Intermediate anhedonia’ group had an increased likelihood of belonging to the low-income group. This could indicate that SES may have an impact in terms of experiencing social anhedonia. It is known that depression and anxiety are more prevalent in people with a low SES [65,66,67,68]. Other studies have also found a clear relationship between social anhedonia and SES [68]. Perhaps these socio-economic differences are what trigger slightly higher anhedonia levels in people who otherwise would have been characterized as belonging to the ‘low anhedonia’ group. However, no results were evident to support this conjecture for the ‘High anhedonia’ group. Nevertheless, the members of the ‘High anhedonia’ group had a higher likelihood of being female than male. Studies on the relationship between gender and social anhedonia have yielded mixed results [68,69,70]; thus, it is unclear whether this higher likelihood of being female in the ‘High anhedonia’ group is simply due to the composition of this specific sample, which seems unlikely as 64% of the sample were male, or whether another factor is mediating this specific link. On the other hand, it could be argued that there is a real chance that this is a true reflection of females being more anhedonic, as it has been widely discovered that the prevalence of depression has increased for women [70,71,72].

In general, a division could be detected between the three segments, wherein the ‘Intermediate anhedonia’ and ‘High anhedonia’ groups shared a great deal of similarities, whereas the ‘Low anhedonia’ group was in many instances depicted as the opposite to the two others. The aspect of ‘Eating alone’ especially emphasized this division, with the ‘Low anhedonia’ segment showing a decreased impact by this aspect, in contrast to the two other segments. As the segments indeed were defined by social anhedonia, it seemed plausible that the social aspects of food pleasure were affected too, and that this would be reflected in the results regarding both the aspect of ‘eating alone’ and ‘eating w. others’. Thus, it appears that anhedonia, even at somewhat moderate levels, affects the perception of food pleasure. This is a key result, as an intermediate or even high anhedonia level is not necessarily related to any mental health issues. This was evident from the results depicted in Figure 2. Thus, it could be speculated that changes in the perception of pleasure from food could be one of the very early signs of anhedonia, potentially leading to changes in appetite and food choices too.

Therefore, further studies into the changes in appetite among people with mental disorders are highly recommended to fully understand how anhedonia level combined with specific mental disorders ultimately affects eating behaviour. Moreover, the repercussions in terms of the perception of wellbeing from food at these levels could possibly inflict a general lack of joy in everyday life, thus also potentially leading to the development of mental diseases such as depression. At the same time, the segmentation analyses proved that the members of both the ‘Intermediate anhedonia’ and ‘High anhedonia’ groups had significantly increased likelihoods of scoring high on both the PHQ-9 and the GAD-7. A clear link between social anhedonia and these mental diseases has thereby been established from this study, and it is evident that no matter the order of causality, suffering from either depression, anxiety, or anhedonia will affect one’s perception of pleasure from food, further implying the very likely possibility of altered food choices and eating behaviours too.

### 4.3. Implications

From this study, new insights into the relationship between anhedonic traits and perceived pleasure from food and eating, as experienced by people suffering from depression and/or anxiety, have been defined. The commonalities of the symptoms experienced and expressed as anhedonia in depression and anxiety states and the possible links to eating behaviour have been defined in previous studies [5,29,56]; however, the direct link shown in the present study has not been investigated previously. Having research on a large sample where both healthy people and people with anhedonic traits were present was a particularly significant aspect of the present study, as it made it possible to control for the significance of whether these health conditions were responsible for the differences expressed in the results of the Food Pleasure Scale. We would recommend that larger population samples are used in such studies to enable a more nuanced interpretation of the results. In addition, the division of three segments based on the level of anhedonia allowed for a detailed examination of the significance of the impact of this condition on the perceived experience of food pleasure. Thus, valuable insights into the food pleasure profiles of people with different levels of social anhedonia have been found for future research in the fields of mental illness, (food) anhedonia, and consumer behaviours.

Depression and anxiety have long been a major global health issue with ever increasing prevalence rates. Furthermore, it is expected that depression will become the top cause of the global burden of disease in 2030 [72]. Discovering new insights on the repercussions of these conditions, therefore, appears to be a necessary step towards making further progress towards both better prevention strategies and treatment options. The insights from the present study can be of great value in the public health sector for use in both the design of new treatments and in terms of the better guidance of patients suffering from these conditions. We suggest that the Food Pleasure Scale can be used as a tool for detecting early signs of depression and/or anxiety and as the basis for a dietary intervention in terms of enhancing wellbeing in everyday life for the prevention of a further onset of depressive disorders. Thus, by alleviating anhedonic symptoms, both mental and physical health have the possibility of increasing too, thereby leading to greater resilience against these mental conditions.

### 4.4. Strengths and Limitations of the Study

The present study contributes to the multicomplex area of understanding the perception of food pleasure, and thereby food choices and eating behaviours too. Through this study, new insights into how people with anhedonic traits experience food and pleasure from food have been discovered. These insights help to understand and unravel some of the effects that anhedonia and mental health diseases can have on the altered eating behaviours, both in people with mild symptoms as well as those with a more severe condition. The sample of this study was a particular strength, as it both represented people with symptoms of depression and anxiety, both conditions known to involve anhedonia, as well as people with no mental health issues and very low levels of anhedonia. Thus, it was possible to compare the results of a ‘healthy’ group and a group with depression, anxiety, and anhedonia symptoms. Indeed, this heightens the strength of the results found. Furthermore, the size of the sample made it possible to conduct a segmentation analysis including three different segments: a ‘Low anhedonia’, ‘Intermediate anhedonia’, and ‘High anhedonia’ group. To the authors’ knowledge, no such study has previously been conducted that focused on the different levels of anhedonia as the basis for a segmentation analysis and profiling. Finally, the scales used to assess MDD, anxiety, and social anhedonia levels are all well-established, well-tested, and validated scales, further adding to the reliability and strength of the study results. The combination of the results of these scales with the Food Pleasure Scale, which can provide new insights into people’s perception of pleasure in relation to food and eating, thus made way for unique discoveries.

This study was an online consumer questionnaire based on subjective self-reported responses. A significant limitation of this method is that its results are dependent on reflection and retrospection, especially for the questions regarding food pleasure. Furthermore, there is the well-known self-report bias of respondents modifying answers for a better fit of their own self-perception [73]. Thus, the results regarding the more personal or sensitive matters, such as those obtained using the PHQ-9 and GAD-7, may have been affected, which could diminish the individual scores of these scales. However, as both of these scales have proven to be highly reliable and are used for medical diagnosis purposes on a global scale, the authors found no reason to doubt the results of these scales. It could be argued that the two conditions are not comparable in terms of their characteristics, severity, and implications on individual life and wellbeing, and thus should not be grouped as one collected group of consumers. Nonetheless, the authors found that for fulfilling the aims of this study, a comparison of people with depression and/or anxiety to people with no signs of these conditions was a fruitful and effective way of receiving insights on people with anhedonic traits. Furthermore, as previous studies have documented that a dominant link between the two conditions is, in fact, anhedonia [18], and since the majority of the sample (*n* = 196, 56%) proved to be suffering from both conditions simultaneously, a merging of the two conditions seemed reasonable. Even though the study was conducted solely on a sample of Chinese consumers, the sample cannot be considered representative of the general Chinese population. This would require a larger sample size and the representation of consumer segments as found in the Chinese population. In addition, the classification used for an intermediate level of social anhedonia group might be arbitrary and should be treated as one of the limitations of the present study. Finally, there was a slight overrepresentation of men in this sample. However, as all data analyses of this study were controlled for age and gender, these socio-demographic factors are not reflected in the profiles of the segments. Nonetheless, due to the exploratory nature of this study, the study can inspire researchers and health professionals towards making further progress in research focusing on the prevention of the repercussions of these mental diseases. A deeper knowledge of what drives the individual perception of food pleasure could be an active tool in the treatment of depression and other health states involving anhedonia.

## 5. Conclusions

This exploratory consumer study has taken us a step closer in terms of understanding the relationship between anhedonia, as expressed by people suffering from depression and anxiety, and the perception of pleasure from food. Thus, the understanding of ‘food pleasure’, as a fairly new concept within the field of sensory and consumer science, has been further explored and new insights have been attained. These very first results on consumers documented to have anhedonic traits showed that food pleasure indeed was affected by social anhedonia, as well as common mental health diseases, depending on the level of anhedonia. Overall, it was found that both ‘healthy’ consumers and consumers with depression and anxiety found pleasure in the sensory modalities of food, as well as the cognitively driven aspects, such as fulfilling one’s needs and expectations and having choices. However, clear differences were detected too, as the depression and anxiety groups showed a reduction in variety and in frequencies of choice with respect to most food-related pleasure aspects.

Moreover, the results from the segmentation analysis based on three different levels of anhedonia (i.e., low anhedonia, intermediate anhedonia, and high anhedonia levels) proved to be fruitful, as a more nuanced insight into specific differences in the perception of food pleasure was obtained. It was found that the ‘Low anhedonia’ group found pleasure in almost all aspects of the Food Pleasure Scale, especially those that reflect the contextually and exploratorily driven sides of pleasure. On the other hand, the aspect of eating alone was not appreciated by this segment. Eating alone was, however, appreciated by both the ‘Intermediate anhedonia’ and ‘High anhedonia’ groups, underpinning the effect of social anhedonia on food pleasure. Furthermore, both of these segments proved that their perceptions of food pleasure in general were affected by anhedonia, as most secondary aspects of the food pleasure scale (except for eating alone) were less likely to be contributary. Interestingly, the profile of the ‘High anhedonia’ group greatly mirrored that of the ‘Intermediate anhedonia’ group, but with amplified results for each aspect. In addition, the more basic aspects of food pleasure, the sensory modalities of food, were also negatively associated with food pleasure for this segment, indicating that this level of anhedonia has reached a point where the food itself starts to bring forth aversive sensations and expectations.

To be able to fully understand the impact of anhedonia levels on eating behaviour, further investigations of the changes in appetite among people with anhedonic traits, such as people with mental disorders, are highly recommended. Thus, a better understanding of the repercussions in terms of the perception of wellbeing through food of consumers affected by mental disorders could likewise be attained, potentially leading to new prevention strategies and treatments of these consumers.

## Figures and Tables

**Table 1:** Characteristics of respondents.

Characteristics	
**n_total_**	1051
Males/females (%)	676 (64%)/375 (36%)
Age (years) *	21.90 ± 4.10 (18–50)
Education (years) *^, 1^	11.33 ± 1.49 (9–13)
Fathers age (years) *	50.02 ± 5.89 (32–76)
Mothers age (years) *	48.33 ± 5.86 (35–74)
Fathers’ education (years) *^, 1^	9.45 ± 3.66 (0–16)
Mothers’ education (years) *^, 1^	8.33 ± 3.64 (0–16)
**Monthly income of whole family, CNY ^2^**	
≤2500 (%)	27 (3%)
2501–5000 (%)	118 (11%)
5001–10,000 (%)	392 (37%)
10,001–20,000 (%)	349 (33%)
≥20,000 (%)	165 (16%)
Mental illness diagnosis (%)	0 (0%)
**Family history of mental illnesses**	
Yes (%)	0 (0%)
No (%)	1017 (97%)
Unsure (%)	34 (3%)
PHQ-9 score *^, 3^	7.68 ± 5.33 (0–27)
No depression (%)	725 (69%)
With depression (%)	326 (31%)
GAD-7 score *^, 4^	5.79 ± 4.87 (0–21)
No anxiety (%)	832 (79%)
With anxiety (%)	219 (21%)
RSAS score *^, 5^	12.52 ± 7.65 (0–40)
Low (%)	537 (51%)
Intermediate (%)	360 (34%)
High (%)	154 (15%)

* Mean ± standard deviation (range), ^1^ Education includes primary school, ^2^ CNY: Chinese Yuan, ^3^ PHQ-9: 9-item Patient Health Questionnaire, ^4^ GAD-7: 7-item General Anxiety Disorder, and ^5^ RSAS: Revised Social Anhedonia Scale.

**Table 2:** Characterization of the three (an)hedonia segments.

	Low Anhedonia	Intermediate Anhedonia	High Anhedonia
**n_total_**	537	360	154
Males/females (%)	348 (65%)/189 (35%)	239 (66%)/121 (34%)	89 (58%)/65 (42%)
Age (years) *	22.05 (±4.25)	21.71 (±3.62)	21.80 (±4.58)
Education (years) *^,^ ^1^	14.68 (±2.15)	14.73 (±0.50)	14.44 (±2.31)
Father’s age (years) *	50.15 (±6.25)	49.68 (±5.24)	50.35 (±5.96)
Mother’s age (years) *	48.41 (±6.14)	48.06 (±5.42)	48.71 (±5.80)
Father’s education (years) *^,^ ^1^	10.26 (±3.46)	9.76 (±3.97)	9.83 (±3.00)
Mother’s education (years) *^,^ ^1^	9.42 (±3.44)	8.93 (±3.92)	8.90 (±3.32)
**Monthly income of whole family—CNY ^2^**			
≤2500 (%)	7 (1%)	16 (4%)	4 (3%)
2501–5000 (%)	59 (11%)	43 (12%)	16 (10%)
5001–10,000 (%)	206 (38%)	124 (34%)	62 (40%)
10,001–20,000 (%)	169 (31%)	127 (35%)	53 (34%)
≥20,000 (%)	96 (18%)	50 (14%)	19 (12%)
**Mental illness diagnosis (%)**	0 (0%)	0 (0%)	0 (0%)
**Family history of mental illnesses**			
Yes (%)	0 (0%)	0 (0%)	0 (0%)
No (%)	527 (98%)	345 (96%)	145 (94%)
Unsure (%)	10 (2%)	15 (4%)	9 (6%)
PHQ-9 score *^, 3^	5.40 (±4.22)	9.22 (±4.95)	12.06 (±5.67)
No depression (%)	457 (85%)	208 (58%)	60 (39%)
With depression (%)	80 (15%)	152 (42%)	94 (61%)
GAD-7 score *^, 4^	3.89 (±3.88)	7.06 (±4.90)	9.41 (±4.93)
No anxiety (%)	488 (91%)	254 (71%)	90 (58%)
With anxiety (%)	49 (9%)	106 (29%)	64 (42%)
RSAS score *^, 5^	6.48 (±3.03)	15.82 (±2.74)	25.83 (±4.41)

* Mean ± standard deviation (range), ^1^ Education includes primary school, ^2^ CNY: Chinese Yuan, ^3^ PHQ-9: 9-item Patient Health Questionnaire, ^4^ GAD-7: 7-item General Anxiety Disorder, and ^5^ RSAS: Revised Social Anhedonia Scale.

## Data Availability

The datasets generated for this study are available upon request made to the corresponding author.

## Supplementary Materials

The following supporting information can be downloaded at: https://www.mdpi.com/article/10.3390/foods11223659/s1, Figure S1: Schematic conceptualization of the key dimensions, items, and behavioral elements involved in the individual food-related pleasure response, allowing for a holistic study of quantitative (level of pleasure) and qualitative (drivers of pleasure) aspects of food-related pleasure; Table S1: Multivariate Regression by odds ratio on all variables for each of the three segments.
