# Supplementary material for: The Relationship between Social Anhedonia and Perceived Pleasure from Food—An Exploratory Investigation on a Consumer Segment with Depression and Anxiety
Source: Foods. 2022 Nov 16;11(22):3659. doi: 10.3390/foods11223659 (PMC9689578; doi:10.3390/foods11223659)
Supplement: Supplementary file 1 [file foods-11-03659-s001.zip › foods-1956568-supplementary.pdf]

Supplementary material

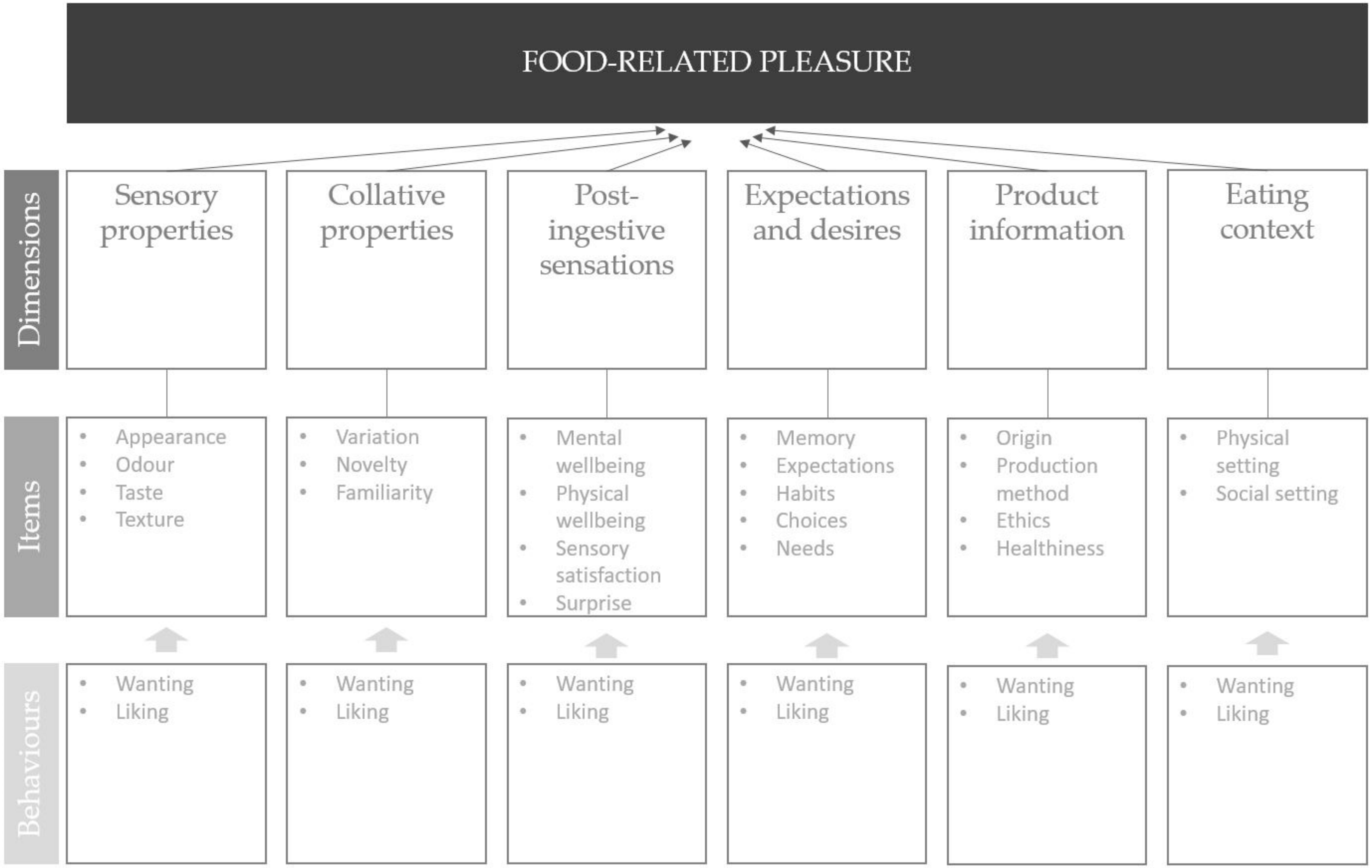

Figure S1. Schematic conceptualisation of the key dimensions, items, and behavioural elements involved in the individual food-related pleasure response, allowing a holistic study of quantitative (level of pleasure) and qualitative (drivers of pleasure) aspects of food-related pleasure. The model is kindly borrowed from Andersen, B. V., Chan, R. C. K., & Byrne, D. V. (2021). A Conceptual Framework for Multi-Dimensional Measurements of Food Related Pleasure—The Food Pleasure Scale. Foods, 10(9), 2044. https://doi.org/10.3390/foods10092044

Table S1. Multivariate Regression by Odd's Ratio on all variables for each of the three segments.

	Low anhedonia n=537		Intermediate anhedonia n=360		High anhedonia n=154	
	**Odd's Ratio (CI)**	**p-value**	**Odd's Ratio (CI)**	**p-value**	**Odd's Ratio (CI)**	**p-value**
**Sociodemographic**						
**Gender (m/f)**						
Male	1		1		1	
Female	0.96 (0.74-1.23)	0.737	0.87 (0.67-1.14)	0.312	1.38 (0.97-1.96)	0.068
**Age**	1.02 (0.99-1.05)	0.214	1.06 (0.99-1.14)	0.284	0.99 (0.95-1.03)	0.749
**Education (years)**[1]	0.99 (0.92-1.05)	0.668	0.99 (0.97-1.02)	0.110	0.93 (0.85-1.02)	0.130
**Fathers age**	1.00 (0.97-1.02)	0.802	1.04 (1.01-1.07)	0.570	1.02 (0.98-1.06)	0.264
**Fathers education**[1]	1.05 (1.01-1.08)	0.017	0.97 (0.93-1.00)	0.086	0.97 (0.92-1.02)	0.288
**Mothers age**	0.99 (0.97-1.02)	0.610	0.99 (0.96-1.02)	0.618	1.03 (0.99-1.07)	0.166
**Mothers education**[1]	1.03 (1.00-1.07)	0.035	0.98 (0.95-1.01)	0.186	0.97 (0.93-1.02)	0.227
**Monthly income of whole family**[2]						
- ≤ 2500 RMB	0.31 (0.12-0.72)	0.010	3.26 (1.48-7.44)	0.004	0.89 (0.25-2.43)	0.840
- 2501-5000 RMB	0.91 (0.6-1.38)	0.659	1.25 (0.81-1.92)	0.317	0.81 (0.43-1.44)	0.489
- 5001-10000 RMB	1		1		1	
- 10001-20000 RMB	0.82 (0.61-1.10)	0.189	1.26 (0.93-1.72)	0.140	0.98 (0.65-1.46)	0.912
- ≥ 20000 RMB	1.22 (0.84-1.276)	0.303	0.96 (0.64-1.43)	0.861	0.70 (0.40-1.21)	0.217
**Health**						
**Mental illness diagnosis**	NA		NA		NA	
**Family hist. mental illnesses**						
- Yes	NA		NA		NA	
- No	1		1		1	
- Unsure	0.36 (0.16-0.74)	0.008	1.66 (0.81-3.33)	0.156	2.18 (0.93-4.66)	0.055
**PHQ-9 score**[3]	0.81 (0.79-0.84)	<0.001	1.09 (1.06-1.11)	<0.001	1.19 (1.15-1.23)	<0.001
**GAD-7 score**[4]	0.82 (0.79-0.85)	<0.001	1.09 (1.06-1.12)	<0.001	1.18 (1.14-1.22)	<0.001
**Pleasure**						
**RSAS score**[5]	NA	0.989	1.09 (1.07-1.11)	<0.001	NA	0.986
**FPS**[6] **– I get pleasure from**						
Memories	2.30 (1.25-4.42)	0.009	0.52 (0.29-0.95)	0.031	0.73 (0.36-1.63)	0.401
Expectations	1.66 (0.96-2.91)	0.073	0.81 (0.47-1.43)	0.462	0.59 (0.31-1.19)	0.118
Needs	1.39 (0.77-2.55)	0.284	0.99 (0.54-1.89)	0.982	0.58 (0.29-1.25)	0.136
Choices	2.17 (1.28-3.78)	0.005	0.73 (0.43-1.23)	0.225	0.49 (0.27-0.91)	0.018
Habits	1.48 (1.02-2.14)	0.038	0.72 (0.50-1.05)	0.083	0.86 (0.53-1.43)	0.544
Ethical values	1.47 (1.05-2.07)	0.026	0.95 (0.67-1.36)	0.771	0.56 (0.37-0.86)	0.007

Product information	1.80 (1.29-2.51)	<0.001	0.63 (0.45-0.88)	0.007	0.76 (0.50-1.18)	0.210
Physical surroundings	1.87 (1.18-3.02)	0.008	0.72 (0.45-1.14)	0.158	0.59 (0.35-1.06)	0.067
Eating with others	2.80 (1.89-4.21)	<0.001	1.19 (0.80-1.79)	0.393	0.19 (0.13-0.29)	<0.001
Eating alone	0.57 (0.44-0.75)	<0.001	1.51 (1.13-2.03)	0.005	1.50 (1.01-2.28)	0.053
Variation	1.93 (1.15-3.32)	0.014	0.77 (0.46-1.31)	0.323	0.51 (0.28-0.97)	0.030
Familiarity	1.67 (1.05-2.70)	0.032	1.05 (0.65-1.74)	0.837	0.42 (0.25-0.73)	0.001
Novelty	2.25 (1.42-3.65)	<0.001	0.73 (0.47-1.16)	0.180	0.45 (0.27-0.77)	0.003
Appearance	1.53 (0.97-2.43)	0.071	0.91 (0.57-1.47)	0.703	0.56 (0.33-1.00)	0.040
Odor	1.26 (0.68-2.36)	0.472	1.19 (0.62-2.39)	0.615	0.54 (0.27-1.17)	0.095
Taste	2.06 (1.08-4.10)	0.031	0.67 (0.36-1.02)	0.221	0.57 (0.28-1.29)	0.148
Texture	1.56 (0.84-2.99)	0.164	0.97 (0.51-1.90)	0.927	0.50 (0.25-1.09)	0.063
Pleased senses	3.59 (1.99-6.87)	<0.001	0.43 (0.25-0.73)	0.002	0.56 (0.30-1.11)	0.077
Physical sensations	2.20 (1.45-3.41)	<0.001	0.58 (0.39-0.88)	0.009	0.64 (0.39-1.10)	0.090
Mental sensations	1.99 (1.22-3.31)	0.007	0.70 (0.43-1.15)	0.150	0.56 (0.32-1.03)	0.051
Surprise	2.24 (1.41-3.63)	<0.001	0.57 (0.36-0.89)	0.014	0.65 (0.38-1.18)	0.138
**FPS[6] – Importance**						
Memories						
- Not very important	0.62 (0.36-1.06)	0.086	1.04 (0.61-1.75)	0.885	1.99 (1.03-3.78)	0.037
- Neither nor	1		1		1	
- Very important	1.49 (1.10-2.02)	0.011	0.65 (0.48-0.89)	0.007	1.00 (0.65-1.58)	0.985
Expectations						
- Not very important	0.65 (0.38-1.08)	0.102	1.03 (0.62-1.72)	0.900	1.84 (0.98-3.39)	0.053
- Neither nor	1		1		1	
- Very important	1.50 (1.11-2.04)	0.009	0.72 (0.53-0.99)	0.042	0.80 (0.53-1.25)	0.317
Needs						
- Not very important	1.00 (0.60-1.68)	0.987	0.72 (0.42-1.24)	0.244	1.54 (0.83-2.82)	0.168
- Neither nor	1		1		1	
- Very important	1.54 (1.13-2.11)	0.007	0.82 (0.59-1.13)	0.223	0.62 (0.41-0.95)	0.026
Choices						
- Not very important	0.83 (0.52-1.32)	0.437	0.73 (0.45-1.17)	0.191	2.08 (1.19-3.63)	0.010
- Neither nor	1		1		1	
- Very important	1.67 (1.24-2.26)	<0.001	0.64 (0.48-1.14)	0.005	0.80 (0.53-1.23)	0.305
Habits						
- Not very important	0.86 (0.57-1.29)	0.455	0.91 (0.60-1.38)	0.656	1.51 (0.89-2.54)	0.120
- Neither nor	1		1		1	
- Very important	1.49 (1.13-1.98)	0.006	0.69 (0.52-0.93)	0.015	0.87 (0.58-1.31)	0.489
Ethical values						
- Not very important	0.68 (0.46-0.99)	0.042	1.09 (0.74-1.61)	0.651	1.76 (1.06-2.94)	0.030

- Neither nor	1		1		1	
- Very important	1.19 (0.89-1.59)	0.106	0.76 (0.56-1.03)	0.075	1.17 (0.76-1.81)	0.485
Product information						
- Not very important	0.73 (0.50-1.06)	0.098	1.24 (0.85-1.80)	0.261	1.20 (0.73-1.93)	0.469
- Neither nor	1		1		1	
- Very important	1.60 (1.20-2.12)	0.001	0.67 (0.50-0.91)	0.009	0.79 (0.53-1.19)	0.253
Physical surroundings						
- Not very important	0.51 (0.32-0.81)	0.005	1.37 (0.87-2.17)	0.171	1.69 (0.97-2.89)	0.059
- Neither nor	1		1		1	
- Very important	1.33 (1.00-1.77)	0.049	0.88 (0.66-1.20)	0.421	0.70 (0.47-1.05)	0.078
Eating with others						
- Not very important	0.51 (0.33-0.76)	0.001	0.89 (0.60-1.32)	0.567	2.84 (1.79-4.53)	<0.001
- Neither nor	1		1		1	
- Very important	1.81 (1.36-2.41)	<0.001	0.68 (0.51-0.92)	0.012	0.57 (0.37-0.88)	0.010
Eating alone						
- Not very important	1.30 (0.96-1.77)	0.090	0.81 (0.58-1.11)	0.194	0.84 (0.51-1.34)	0.465
- Neither nor	1		1		1	
- Very important	0.76 (0.57-1.01)	0.061	0.96 (0.71-1.29)	0.193	1.77 (1.20-2.63)	0.004
Variation						
- Not very important	0.77 (0.45-1.31)	0.344	0.85 (0.50-1.45)	0.563	1.86 (0.98-3.49)	0.056
- Neither nor	1		1		1	
- Very important	1.50 (1.09-2.07)	0.014	0.74 (0.53-1.03)	0.070	0.78 (0.51-1.24)	0.279
Familiarity						
- Not very important	0.85 (0.54-1.33)	0.481	0.92 (0.58-1.45)	0.731	1.47 (0.83-2.58)	0.176
- Neither nor	1		1		1	
- Very important	1.45 (1.08-1.94)	0.014	0.74 (0.54-1.00)	0.047	0.84 (0.56-1.28)	0.398
Novelty						
- Not very important	0.76 (0.49-1.19)	0.232	1.24 (0.79-1.93)	0.347	1.10 (0.62-1.90)	0.732
- Neither nor	1		1		1	
- Very important	1.58 (1.19-2.09)	0.001	0.76 (0.57-1.02)	0.071	0.66 (0.45-0.97)	0.033
Appearance						
- Not very important	0.60 (0.39-0.92)	0.020	1.03 (0.66-1.59)	0.893	2.28 (1.31-3.96)	0.003
- Neither nor	1		1		1	
- Very important	1.06 (0.79-1.42)	0.696	0.89 (0.66-1.22)	0.468	1.11 (0.72-1.73)	0.663
Odor						
- Not very important	0.51 (0.29-0.90)	0.020	1.35 (0.77-2.35)	0.289	1.80 (0.91-3.51)	0.087
- Neither nor	1		1		1	
- Very important	1.08 (0.78-1.50)	0.630	0.99 (0.70-1.40)	0.937	0.87 (0.56-1.40)	0.559

Taste						
- Not very important	0.72 (0.37-1.35)	0.308	1.04 (0.55-1.93)	0.906	1.58 (0.71-3.39)	0.250
- Neither nor	1		1		1	
- Very important	1.58 (1.09-2.28)	0.015	0.67 (0.47-1.02)	0.037	0.85 (0.52-1.44)	0.516
Texture						
- Not very important	0.42 (0.21-0.83)	0.015	0.66 (0.35-1.23)	0.193	5.76 (2.68-12.85)	<0.001
- Neither nor	1		1		1	
- Very important	1.67 (1.15-2.44)	0.008	0.51 (0.35-0.75)	<0.001	1.35 (0.76-2.59)	0.332
Pleased senses						
- Not very important	0.49 (0.25-0.95)	0.040	0.81 (0.45-1.47)	0.495	3.30 (1.62-6.78)	0.001
- Neither nor	1		1		1	
- Very important	2.03 (1.42-2.94)	<0.001	0.48 (0.34-0.69)	<0,001	0.98 (0.59-1.71)	0.953
Physical sensations						
- Not very important	0.37 (0.21-0.62)	<0.001	1.31 (0.80-2.13)	0.278	2.70 (1.50-4.84)	<0.001
- Neither nor	1		1		1	
- Very important	1.32 (0.98-1.78)	0.070	0.76 (0.56-1.03)	0.079	0.95 (0.62-1.50)	0.830
Mental sensations						
- Not very important	0.57 (0.34-0.96)	0.037	1.17 (0.71-1.93)	0.531	1.78 (0.95-3.31)	0.068
- Neither nor	1		1		1	
- Very important	1.62 (1.17-2.25)	0.004	0.65 (0.47-0.91)	0.013	0.83 (0.53-1.32)	0.415
Surprise						
- Not very important	0.35 (0.21-0.55)	<0.001	1.63 (1.04-2.55)	0.033	2.31 (1.33-4.00)	0.003
- Neither nor	1		1		1	
- Very important	1.08 (0.81-1.45)	0.604	0.96 (0.70-1.31)	0.779	0.92 (0.60-1.44)	0.713

[1]Education includes primary school, [2]CNY: Chinese Yuan, [3]PHQ-9: 9-item Patient Health Questionnaire, [4]GAD-7: 7-item General Anxiety Disorder, [5]RSAS: Revised Social Anhedonia Scale, [6]FPS: Food Pleasure Scale
